# A qualitative assessment of factors affecting nursing home caregiving staff experiences during the COVID-19 pandemic

**DOI:** 10.1371/journal.pone.0260055

**Published:** 2021-11-15

**Authors:** Rachel L. Snyder, Laura E. Anderson, Katelyn A. White, Stephanie Tavitian, Lucy V. Fike, Heather N. Jones, Kara M. Jacobs-Slifka, Nimalie D. Stone, Ronda L. Sinkowitz-Cochran

**Affiliations:** Division of Healthcare Quality Promotion, Centers for Disease Control and Prevention, Atlanta, Georgia, United States of America; University of Haifa, ISRAEL

## Abstract

**Background:**

A large portion of COVID-19 cases and deaths in the United States have occurred in nursing homes; however, current literature including the frontline perspective of staff working in nursing homes is limited. The objective of this qualitative assessment was to better understand what individual and facility level factors may have contributed to the impact of COVID-19 on Certified Nursing Assistants (CNAs) and Environmental Services (EVS) staff working in nursing homes.

**Methods:**

Based on a simple random sample from the National Healthcare Safety Network (NHSN), 7,520 facilities were emailed invitations requesting one CNA and/or one EVS staff member for participation in a voluntary focus group over Zoom. Facility characteristics were obtained via NHSN and publicly available sources; participant demographics were collected via SurveyMonkey during registration and polling during focus groups. Qualitative information was coded using NVIVO and Excel.

**Results:**

Throughout April 2021, 23 focus groups including 110 participants from 84 facilities were conducted homogenous by participant role. Staffing problems were a recurring theme reported. Participants often cited the toll the pandemic took on their emotional well-being, describing increased stress, responsibilities, and time needed to complete their jobs. The lack of consistent and systematic guidance resulting in frequently changing infection prevention protocols was also reported across focus groups.

**Conclusions:**

Addressing concerns of low wages and lack of financial incentives may have the potential to attract and retain employees to help alleviate nursing home staff shortages. Additionally, access to mental health resources could help nursing home staff cope with the emotional burden of the COVID-19 pandemic. These frontline staff members provided invaluable insight and should be included in improvement efforts to support nursing homes recovering from the impact of COVID-19 as well as future pandemic planning.

## Introduction

A large portion of COVID-19 cases and deaths in the United States have occurred in nursing homes and other long-term care settings, with over 1.3 million confirmed cases and over 137,000 confirmed deaths among residents and staff as of September 12, 2021 [1]. During the pandemic, nursing homes have also experienced shortages of both personal protective equipment (PPE) and staff, potentially affecting their ability to safely provide care [2]. In addition to the risk of both contracting and spreading SARS-CoV-2, the virus causing COVID-19, the mental health of nursing home staff may also be affected by the pandemic, as several studies have illustrated symptoms of anxiety, depression, and post-traumatic stress in frontline healthcare personnel during the pandemic [3–5]. As staff are likely important contributors to the transmission of SARS-CoV-2 in nursing home settings [6], more knowledge is needed regarding the experiences of nursing home staff during the COVID-19 pandemic.

Despite their important role in both the care of residents and preventing spread of SARS-CoV-2, current literature that includes the frontline perspective of Certified Nursing Assistants (CNA) and Environmental Services (EVS) staff members (also referred to as housekeeping) working in nursing homes is limited. The objective of this qualitative assessment was to better understand what individual and facility-level factors may have contributed to the impact of the COVID-19 pandemic in nursing homes by examining the perceptions of CNAs and EVS staff regarding COVID-19 prevention efforts and self-reported behaviors and beliefs.

## Methods

### Pilot

Four pilot focus groups were conducted with a total of 30 CNAs from Genesis Healthcare nursing home facilities from February 3–12, 2021. Pilot data were used to standardize discussion and polling questions and refine recruitment processes for the expanded focus groups. Data collected during the pilot are not included in this manuscript.

### Recruitment

Facilities were selected based on a simple random sample of the 15,351 long-term care facilities actively reporting to the National Healthcare Safety Network (NHSN) as of March 23, 2021. NHSN Administrators from 7,520 facilities were emailed invitations requesting one CNA and/or one EVS staff member from each facility to voluntarily participate in a focus group.

### Focus groups

Focus groups were conducted homogenous by participant role (CNAs separate from EVS) and were offered during weekdays and weekends, with morning, afternoon, and night sessions to accommodate differing shifts. On average, focus groups ranged in length from 35–50 minutes and varied in size from one to ten participants. To encourage open sharing, all responses provided by participants were confidential and no individual comments from focus groups were shared with supervisors or nursing homes where participants were employed.

### Data sources

Facility characteristics were obtained via NHSN, the National Center for Health Statistics (NCHS) Urban-Rural Classification Scheme [7] and the Centers for Disease Control and Prevention (CDC) Social Vulnerability Index (SVI) [8] based on facility county. Chi-square tests were used to compare the distribution of selected variables (urban/rural facility location, SVI quartile, facility bed size quartile, and facility ownership) between participant facilities and the general population of nursing homes actively reporting into NHSN, with p values < 0.05 considered statistically significant. Analyses were conducted using SAS version 9.4 software (SAS Inc., Cary, NC, USA) for Windows. Individual participant demographics were obtained via a voluntary and anonymous SurveyMonkey during registration, as well as voluntary polling on the Zoom platform during the focus groups. As such, individual demographic data were not provided for all participants, and the demographics summaries may include data from individuals who registered but did not participate in the focus groups. Polling on the Zoom platform was also used to obtain voluntary responses to questions regarding perceived risk of getting COVID-19 in the facility (at the beginning of the pandemic and at the time of focus groups), greatest barrier/challenge to preventing COVID-19 in the facility (at the beginning of the pandemic and at the time of focus groups), and preferred channels of communication. The remaining information was obtained during open discussion with the use of a standardized script and questions guided by a trained facilitator regarding facility strengths and weaknesses, changes in job responsibilities, what the participants wish they had known at the start of the pandemic, what they are most worried/concerned about moving forward, and other topics specific to COVID-19. These data were qualitatively coded by question using an immersion and crystallization technique and summarized using NVIVO and Excel across a team of trained coders to ensure reliability [9]. No tests for statistical significance were performed among discussion responses or individual participant demographics.

All responses were provided voluntarily and, due to the open discussion format, not every participant provided a response to every question nor were they directly asked to do so during the focus groups. Percentages presented are calculated based on the total number of participants that provided a response to the individual question, not the total number of focus group participants. Participants’ responses were categorized to each individual code only once; however, responses may be categorized to more than one code. Therefore, percentages within a question may sum to over 100% and may not sum to the percentages for the broader convergent themes presented in text. This activity was reviewed by CDC and was conducted consistent with applicable federal law and CDC policy (See e.g., 45 C.F.R. part 46, 21 C.F.R. part 56; 42 U.S.C. §241(d); 5 U.S.C. §552a; 44 U.S.C. §3501 et seq.). Participants provided verbal consent prior to the start of the focus groups. Per determination by the CDC’s National Center for Emerging and Zoonotic Infectious Diseases (NCEZID) Human Subjects Advisor, this qualitative assessment does not meet the definition of research under 45 C.F.R. 46.102(l) and IRB review is not required. NCEZID’s determination holds that the project did not require submission to CDC’s Human Research Protection Office as granting authority is delegated to the CDC Centers, Institutes, and Offices under CDC Policies SSA-2010-01 and SSA-2010-02.

## Results

### Demographics

Throughout April 2021, 23 focus groups were held including 110 participants from 84 nursing home facilities across 34 states. Twelve of the focus groups were held for CNAs (51 participants total) and 11 for EVS staff (59 participants total). Of the 84 participating nursing homes, 73% were located in urban areas and 51% were for-profit facilities (Table 1). When a chi-square test was used to compare the distribution of selected variables between the 84 participant facilities and the general population of nursing homes actively reporting into NHSN, participant facilities had a greater proportion of non-profit ownership, and a smaller proportion of for-profit ownership when compared with the general population of nursing homes (Table 1). A greater percentage of participant facilities were in the second quartile (low/moderate range) of social vulnerability. There were no significant differences in the distributions of bed size or urban/rural facility locations.

**Table 1:** Facility characteristics.

	Non-Participant Facilities (N = 15,267)	Participant Facilities (N = 84)	
	N (%)	N (%)	p-value*
Location Type^a^			
**Rural**	4,276 (28.0%)	23 (27.4%)	**0.90**
**Urban**	10,991 (72.0%)	61 (72.6%)	
Social Vulnerability Index Quartiles (n = 15,344)^b^			
**First Quartile: Low Vulnerability**	3,100 (20.3%)	17 (20.2%)	**0.0136**
**Second Quartile: Low/Moderate**	3,999 (26.2%)	34 (40.5%)	
**Third Quartile: Moderate/High**	4,797 (31.4%)	16 (19.0%)	
**Fourth Quartile: High Vulnerability**	3,364 (22.0%)	17 (20.2%)	
Ownership^c^			
**For profit**	10,726 (70.3%)	43 (51.2%)	**0.0007**
**Government**	976 (6.4%)	8 (9.5%)	
**Non-profit**	3,565 (23.4%)	33 (39.3%)	
Bed Size Quartiles^c^			
**First Quartile: <65 Beds**	3,813 (25.0%)	21 (25.0%)	**0.96**
**Second Quartile: 65–98 Beds**	3,441 (22.5%)	20 (23.8%)	
**Third Quartile: 99–126 Beds**	4,176 (27.4%)	21 (25.0%)	
**Fourth Quartile: >126 Beds**	3,837 (25.1%)	22 (26.2%)	

a. Defined using National Center for Healthcare Statistics Rural-Urban Classification Scheme (https://www.cdc.gov/nchs/data_access/urban_rural.htm#2013_Urban-Rural_Classification_Scheme_for_Counties) with Urban = Counties coded 1–4 and Rural = Counties coded 5–6.

b. Data from CDC Social Vulnerability Index 2018 County Overall Percentile Rankings (https://www.atsdr.cdc.gov/placeandhealth/svi/index.html). CDC SVI uses 15 census variables, including information on Socioeconomic Status, Household Composition & Disability, Minority Status & Language, & Housing Type & Transportation to identify communities that are most likely to need support before, during, and after a hazardous event. Percentile ranking values range from 0 to 1, with higher values indicating greater vulnerability. Seven non-participant facilities in Guam and Puerto Rico did not have data for Social Vulnerability Index.

c. Data from the National Healthcare Safety Network (NHSN).

*Conducted using chi-square test.

The majority of participants answering demographic questions at the time of focus group registration were White (68%) and identified as female (89%) with an average age of 43 years (Table 2). Since the beginning of the pandemic, 39% had tested positive for COVID-19, 75% were fully vaccinated with a COVID-19 vaccine at the time of the focus group, and most (95%) were employed directly through their nursing home facilities. The majority (87%) of participants answering Zoom polling questions during the focus groups reported working day shift, 44% reported having over 10 years of work experience in total, and half (52%) reported at least five years of experience at their current facility.

**Table 2:** Participant demographics.

Age of Participants (years)	Overall (N = 103)	CNA (N = 52)	EVS (N = 51)
Mean	43	40	46
Range	20–63	20–63	23–62
**Race**	**Overall (N = 111)**	**CNA (N = 54)**	**EVS (N = 57)**
White	75 (68%)	37 (69%)	38 (67%)
Black or African American	21 (19%)	10 (19%)	11 (19%)
Asian	4 (4%)	3 (6%)	1 (2%)
American Indian or Alaska Native	1 (1%)	0 (0%)	1 (2%)
Pacific Islander	1 (1%)	0 (0%)	1 (2%)
Do Not Know/Unsure	1 (1%)	0 (0%)	1 (2%)
Other	8 (7%)	4 (7%)	4 (7%)
**Hispanic, Latino/Latina, or Spanish Origin**	**Overall (N = 113)**	**CNA (N = 54)**	**EVS (N = 59)**
Yes	13 (12%)	6 (11%)	7 (12%)
No	100 (88%)	48 (89%)	52 (88%)
**Gender**	**Overall (N = 114)**	**CNA (N = 55)**	**EVS (N = 59)**
Female	101 (89%)	50 (91%)	51 (86%)
Male	13 (11%)	5 (9%)	8 (14%)
**Tested Positive for COVID-19**	**Overall (N = 114)**	**CNA (N = 55)**	**EVS (N = 59)**
Yes	44 (39%)	24 (44%)	20 (34%)
No	70 (61%)	31 (56%)	39 (66%)
**Vaccination Status**	**Overall (N = 114)**	**CNA (N = 55)**	**EVS (N = 59)**
Yes, Fully Vaccinated	85 (75%)	34 (62%)	51 (86%)
Yes, Partially Vaccinated	2 (2%)	2 (4%)	0 (0%)
No, But Planning to Receive Vaccine	5 (4%)	4 (7%)	1 (2%)
No, Have Not Decided Whether to Receive Vaccine	9 (8%)	5 (9%)	4 (7%)
No, Do Not Plan to Receive Vaccine	13 (11%)	10 (18%)	3 (5%)
**Employment Status**	**Overall (N = 114)**	**CNA (N = 55)**	**EVS (N = 59)**
Employed Directly by the Nursing Home Facility	108 (95%)	55 (100%)	53 (90%)
Employed Through a Contracting Company (Agency)	6 (5%)	0 (0%)	6 (10%)
**When Do You Most Frequently Work?**	**Overall (N = 84)**	**CNA (N = 39)**	**EVS (N = 45)**
Day Shift (During the Week)	73 (87%)	29 (74%)	44 (98%)
Night Shift (During the Week)	3 (4%)	3 (8%)	0 (0%)
Evening Shift (During the Week)	8 (10%)	7 (18%)	1 (2%)
Weekend Only	0 (0%)	0 (0%)	0 (0%)
**Years of Experience Total**	**Overall (N = 84)**	**CNA (N = 39)**	**EVS (N = 45)**
0–1 Years	6 (7%)	2 (5%)	4 (9%)
2–5 Years	23 (27%)	7 (18%)	16 (36%)
6–10 Years	18 (21%)	7 (18%)	11 (24%)
10+ Years	37 (44%)	23 (59%)	14 (31%)
**Years of Experience in Current Facility**	**Overall (N = 84)**	**CNA (N = 39)**	**EVS (N = 45)**
<1 Year	10 (12%)	3 (8%)	7 (16%)
1–2 Years	16 (19%)	6 (15%)	10 (22%)
3–4 Years	14 (17%)	6 (15%)	8 (18%)
>5 Years	44 (52%)	24 (62%)	20 (44%)

### Perceived risk of getting COVID-19

When answering a Zoom poll of their perceived risk of getting COVID-19 at their facility on a scale of one (“Not at All”) to ten (“To a Great Extent”), the responses among participants were more evenly distributed when asked about their perceived risk of getting COVID-19 in their facility at the beginning of the pandemic (30% reporting 1, 2, or 3; 40% reporting 4, 5, 6, or 7; 30% reporting 8, 9, or 10) than compared with at the time of the focus group (79% reporting 1, 2, or 3; 17% reporting 4, 5, 6, or 7; 4% reporting 8, 9, or 10) (S1 Fig). When asked to discuss where they felt more at risk of getting COVID-19, almost 75% of participants mentioned feeling more at risk outside of the facility, often comparing the precautions taken at their workplace with the lack of precautions and unknown COVID status among those they encountered outside the facility. In the words of one participant, they felt more at risk “*Outside the facility*, *because we can’t make those people out there obey the rules that’s going to keep [COVID] under control*.”

### Changes in duties and responsibilities of nursing home caregiving staff

When asked how their job responsibilities or duties had changed due to the COVID-19 pandemic, 68% of participants who responded reported performing tasks beyond their scope of work and added responsibilities, 62% reported an increase in time required to complete tasks, and 27% reported added pressures; 7% reported no changes in their responsibilities. Specific changes reported, as shown in Table 3, included the new responsibility of rule and protocol enforcement, as described by one staff member “*We had to come in long days and screen everybody as well as keep a closer eye on interactions between families*,*”* and additional cleaning and disinfection of high-touch surfaces with one participant stating that they “*just clean continuously*.” Participants specifically reported an increase in time required to complete tasks due to frequent donning and doffing of additional PPE and staffing shortages; in the words of one staff member “*that was difficult*, *being short-staffed when actually we need to bump up the disinfection and sanitation and having less people to do it*.” Participants also reported added pressures specifically from the increased stress and anxiety of their job, describing that “*You’re not only worried about yourself and your residents*, *but you’re worried about bringing it home as well*.” Other changes expressed by participants included frequent changes in policies and protocols, for example, “*policies would change on a daily basis*, *so it was a problem*. *It was confusing for most of the staff*.” Additionally, staff reported consideration for the mental health of residents, as one participant indicated, “*we’re kind of running emotional support too*, *trying to be there for the residents while trying to take care of everything else*.”

**Table 3:** Frequency of qualitative codes by theme: How have your job responsibilities changed because of COVID-19?.

Code§	Frequency (%*)		
	Overall (N = 73)	CNA (N = 38)	EVS (N = 35)
**Tasks Beyond Scope of Work and Added Responsibilities**			
*Tasks Beyond Scope of Work*			
Rule and Protocol Enforcement (Screening, Distancing, etc.)	8 (11%)	4 (11%)	4 (11%)
Moving Residents Within Facility	5 (7%)	2 (5%)	3 (9%)
Cleaning and Disinfection	5 (7%)	3 (8%)	2 (6%)
Non-Clinical Care of Residents Like Styling Hair or Bringing Food	5 (7%)	4 (11%)	1 (3%)
Laundry	2 (3%)	1 (3%)	1 (3%)
Responsible for Patients Transferred from Overflowing Hospitals	2 (3%)	2 (5%)	0 (0%)
Administering Medications and Providing Treatments to Residents	2 (3%)	2 (5%)	0 (0%)
Transporting Residents Outside of Facility (Medical Appointments)	1 (1%)	1 (3%)	0 (0%)
Transporting Deceased Residents to Morgue	1 (1%)	0 (0%)	1 (3%)
Maintenance	1 (1%)	0 (0%)	1 (3%)
Temporary Course to Assist Aides with Residents	1 (1%)	0 (0%)	1 (3%)
*Added (Increased) Responsibilities*			
Additional Cleaning and Disinfection of High-touch Surfaces	26 (36%)	5 (13%)	21 (60%)
Additional Laundry	3 (4%)	0 (0%)	3 (9%)
**Increase in Time Required to Complete Tasks**			
Additional PPE or Frequent Donning and Doffing Required	20 (27%)	8 (21%)	12 (34%)
Staff Shortages	15 (21%)	9 (24%)	6 (17%)
Taking More Precautions	9 (12%)	5 (13%)	4 (11%)
Frequent Changes in Protocols/Infection Control Procedures	7 (10%)	4 (11%)	3 (9%)
Increased Workload Overall	4 (5%)	3 (8%)	1 (3%)
Longer Hours or Shifts	4 (5%)	2 (5%)	2 (6%)
Everything in General Takes Longer	4 (5%)	2 (5%)	2 (6%)
More Handwashing	3 (4%)	2 (5%)	1 (3%)
Responsible for More Residents	3 (4%)	3 (8%)	0 (0%)
**Added Pressures**			
Increased Stress and Anxiety to Job	16 (22%)	9 (24%)	7 (20%)
Becoming Like Family to Residents	4 (5%)	4 (11%)	0 (0%)
Consideration of Residents’ Mental Health	2 (3%)	2 (5%)	0 (0%)
**Other**			
No Change in Responsibilities	5 (7%)	4 (11%)	1 (3%)

* All responses were provided voluntarily and, due to the open discussion format, not every participant provided a response to every question. All summaries of qualitatively coded data are based upon only those responses received throughout the discussion. Therefore, percentages presented are calculated based on the total number of participants that provided a response to the individual question, not the total number of focus group participants. Participants’ responses were categorized to each individual code only once, however, responses may be categorized to more than one code. Therefore, percentages within a question may sum to over 100% and may not sum to the percentages for broader convergent themes presented in text. No statistical tests to compare responses by CNA and EVS participants were performed.

**§** Select quotes which operationalize each code may be found in the (S1 Table).

### Perceived barriers to preventing COVID-19 in facility

When answering a Zoom poll of the one greatest barrier to preventing COVID-19 in their facility, the most common barriers participants reported at the beginning of the pandemic were staffing shortages (30%), followed by lack of training or education (20%), lack of PPE (13%), staff disbelief (staff not believing COVID-19 was a problem) (12%), frequent staff turnover (4%) and limited COVID-19 testing (4%); 6% of participants selected that their facility had no barriers to preventing COVID-19 at the beginning of the pandemic and 12% selected the option of other greatest barrier (e.g., “*the Unknown*”).When answering the same question at the time of the focus groups, the greatest barrier to preventing COVID-19 in their facility also was staffing shortages (48%), followed by frequent staff turnover (13%), staff disbelief (8%), lack of leadership support (2%), lack of PPE (1%), and lack of training or education (1%); 15% reported no barriers to preventing COVID-19 at the time of the focus groups and 11% selected the option of other greatest barrier (e.g., opening for visitation).

### Considerations for what nursing home facilities could improve on

When asked what their nursing home could improve on, the most convergent themes reported by participants in the discussion were to improve staffing (33% of respondents), improve infection prevention practices (29%), and improve organizational culture (19%), while 31% of respondents reported no areas for improvement in their facility and that the facility did the best they could in the circumstances. Of note, participants specifically mentioned their facilities could improve by mitigating staffing shortages, including that “*if we had more hands on*, *it could have prevented a lot of things that happened due to COVID*”, in addition to providing incentive payments, as stated by one participant “*If we would have gotten more hazard pay for everyone and not just the people who worked in the COVID [unit]*, *people would have shown up for work more”*, limiting frequency of changes to protocols or guidance (i.e., *“We were constantly changing things employee wise*, *changing things resident wise*”) and improving communication within the facility, as described by one participant, “*Sometimes they don’t give us all the information… sometimes we have no idea what’s going on*” (Table 4).

**Table 4:** Frequency of qualitative codes by theme: What can your nursing home improve on?.

Code^§^	Frequency (%*)		
	Overall (N = 78)	CNA (N = 38)	EVS (N = 40)
**Improve Staffing**			
Mitigate Staffing Shortages	18 (23%)	12 (32%)	6 (15%)
Provide Incentives or Hazard Pay	5 (6%)	3 (8%)	2 (5%)
Hire In-House Staff (Instead of Agency Staff)	3 (4%)	0 (0%)	3 (8%)
**Improve Infection Prevention Practices**			
Limit Constant Changes in Protocols and Guidelines	5 (6%)	1 (3%)	4 (10%)
Enhance Visitor Screening	4 (5%)	1 (3%)	3 (8%)
Ensure Consistent Enforcement of Protocols Across Staff (e.g., Wearing PPE Correctly)	4 (5%)	3 (8%)	1 (3%)
Ensure Regular, Adequate Cleaning and Access to Cleaning Products	4 (5%)	0 (0%)	4 (10%)
Ensure Sufficient PPE	2 (3%)	0 (0%)	2 (5%)
Implement Mask Wearing Sooner	2 (3%)	0 (0%)	2 (5%)
Increase Testing of Staff and Residents	2 (3%)	0 (0%)	2 (5%)
Provide Education/Training to Staff	2 (3%)	1 (3%)	1 (3%)
Dedicate Staff to COVID-19 Units Only	1 (1%)	1 (3%)	0 (0%)
Do Not Open for Visitation Too Soon	1 (1%)	1 (3%)	0 (0%)
Restrict Visitors Earlier in Pandemic	1 (1%)	0 (0%)	1 (3%)
**Improve Organizational Culture/Morale**			
Improve Communication Within Facility	5 (6%)	3 (8%)	2 (5%)
Don’t Expect More Work Done in Same Amount of Time	4 (5%)	0 (0%)	4 (10%)
Improve Organization and Preparation	2 (3%)	1 (3%)	1 (3%)
Promote Teamwork and Accountability Among Staff	2 (3%)	2 (5%)	0 (0%)
Show Appreciation and Compassion to Staff	2 (3%)	1 (3%)	1 (3%)
Support Staff Despite Outside Pressures and Politics	2 (3%)	2 (5%)	0 (0%)
**No Areas for Improvement**			
None (*Our Facility Did the Best They Could*, *Pandemic was New for All*)	24 (31%)	15 (39%)	9 (23%)

* All responses were provided voluntarily and, due to the open discussion format, not every participant provided a response to every question. All summaries of qualitatively coded data are based upon only those responses received throughout the discussion. Therefore, percentages presented are calculated based on the total number of participants that provided a response to the individual question, not the total number of focus group participants. Participants’ responses were categorized to each individual code only once, however, responses may be categorized to more than one code. Therefore, percentages within a question may sum to over 100% and may not sum to the percentages for broader convergent themes presented in text. No statistical tests to compare responses by CNA and EVS participants were performed.

**§** Select quotes which operationalize each code may be found in the (S2 Table).

### Advice/what do you wish you would have known

In a discussion about what participants wish they would have known at the beginning of the pandemic and advice they would give to other CNAs or EVS staff members about COVID-19 in nursing homes, the most common responses were that staff wish they would have known the magnitude of the pandemic (e.g., length, seriousness, transmissibility; including *“I wish from the get-go I would have realized that it was as bad as what they were saying”*), along with advice to use PPE, to treat residents like family, and to wash your hands (Table 5). As expressed by participants, it is essential *“just to make sure that staff are wearing that PPE right*, *make sure you’re washing your hands*. *Have respect for everybody that’s around because it’s not just stressful for you*, *it’s stressful for everybody*. *And if nothing else*, *more stressful for the residents”* and *“to just remember they [the residents] don’t get to go home*. *They live here… and we are their family*, *their friends*, *their husbands*, *their wives…it’s a very serious job*.*”* In addition, one EVS staff member also emphasized *“the importance of our role in keeping things at bay*. *We’re not ancillary employees when it comes to COVID*.*”*

**Table 5:** Frequency of qualitative codes by theme: What do you wish you would have known? what piece of advice would you share with another CNA/EVS staff member about COVID-19?.

Codes§	Frequency (%*)		
	Overall (N = 103)	CNA (N = 50)	EVS (N = 53)
**Magnitude of Pandemic (Length, Seriousness, Transmissibility)**	**26 (25%)**	**11 (22%)**	**15 (28%)**
**Use PPE**	**25 (24%)**	**13 (26%)**	**12 (23%)**
Wear PPE	11 (11%)	8 (16%)	3 (6%)
*Wear PPE Correctly*	*6 (6%)*	*5 (10%)*	*1 (2%)*
Wear Mask	14 (14%)	5 (10%)	9 (17%)
*Wear Mask Correctly*	*3 (3%)*	*1 (2%)*	*2 (4%)*
**Treat Residents Like Family**	**22 (21%)**	**18 (36%)**	**4 (8%)**
**Wash Your Hands**	**22 (21%)**	**10 (20%)**	**12 (23%)**
**Stay Vigilant**	**17 (17%)**	**6 (12%)**	**11 (21%)**
Don’t Let Your Guard Down	8 (8%)	4 (8%)	4 (8%)
Be Careful	8 (8%)	2 (4%)	6 (11%)
Keep Protocols in Place After Outbreak	1 (1%)	0 (0%)	1 (2%)
**Follow IP Guidelines/Precautions**	**15 (15%)**	**6 (12%)**	**9 (17%)**
**Have Emotional Resilience**	**13 (13%)**	**9 (18%)**	**4 (8%)**
**Clean/Disinfect**	**10 (10%)**	**1 (2%)**	**9 (17%)**
Clean Isolation Rooms Last/Don’t Move Isolation Carts Away From COVID-19 Rooms	2 (2%)	0 (0%)	2 (4%)
**Protect Yourself Outside Facility**	**9 (9%)**	**3 (6%)**	**6 (11%)**
**Just Show Up**	**7 (7%)**	**3 (6%)**	**4 (8%)**
**Take Care of Yourself/Stay Healthy**	**5 (5%)**	**1 (2%)**	**4 (8%)**
**Work Together**	**5 (5%)**	**1 (2%)**	**4 (8%)**
**Stay Home if Sick**	**4 (4%)**	**0 (0%)**	**4 (8%)**
**Train, Educate, Prepare**	**4 (4%)**	**2 (4%)**	**2 (4%)**
**Don’t Move Residents Around**	**2 (2%)**	**0 (0%)**	**2 (4%)**
**Warn that Supplies Would Run Out**	**2 (2%)**	**0 (0%)**	**2 (4%)**
**Buy Stock in Alcohol**	**1 (1%)**	**1 (2%)**	**0 (0%)**
**Facilities Shouldn’t Overreact, Overdo it**	**1 (1%)**	**0 (0%)**	**1 (2%)**
**Communicate (Have Adequate Communication)**	**1 (1%)**	**0 (0%)**	**1 (2%)**
**Stay on Floor if Working COVID Unit**	**1 (1%)**	**0 (0%)**	**1 (2%)**
**I Don’t Know**	**1 (1%)**	**1 (2%)**	**0 (0%)**

* All responses were provided voluntarily and, due to the open discussion format, not every participant provided a response to every question. All summaries of qualitatively coded data are based upon only those responses received throughout the discussion. Therefore, percentages presented are calculated based on the total number of participants that provided a response to the individual question, not the total number of focus group participants. Participants’ responses were categorized to each individual code only once, however, responses may be categorized to more than one code. Therefore, percentages within a question may sum to over 100% and may not sum to the percentages for broader convergent themes presented in text. No statistical tests to compare responses by CNA and EVS participants were performed.

**§** Select quotes which operationalize each code may be found in the (S3 Table).

### Concerns and needs of staff moving forward

When participants were asked what they were most worried or concerned about related to COVID-19 in the nursing home moving forward, the most convergent themes mentioned in discussion were fear of experiencing another COVID-19 outbreak (69% of respondents), concerns about the mental wellbeing of staff (26%) and residents (17%), and concerns about staffing capacity and future workforce development (8%); 6% of respondents reported they had no concerns moving forward. As shown in Table 6, specific concerns included fear of COVID-19 coming back into the facility, with one participant describing that they are “*afraid that it’s going to come back into our facility with a force*,*”* along with concerns about complacency in COVID-19 prevention practices and the continued emergence of COVID-19 variants. Concerns about mental well-being specifically included staff emotional strain and anxieties and the effects of social isolation on residents, with one EVS staff member stating, “*To see the heartbreak [of our residents]… it’s heart wrenching to watch… And then [to have to] take that home [as staff members]*. *That’s really difficult*. *For housekeeping especially… We don’t really have all of those tools in our toolbox*. *That mental health*, *being able to process that*, *and not take work home all the time*. *That’s difficult*. *That’s real difficult*.” Additionally, participants expressed concerns about staffing shortages with one participant describing they *“hope that the staffing gets better*. *No one wants to come and work in a nursing home*.*”*

**Table 6:** Frequency of qualitative codes by theme: What are you most concerned about moving forward?.

Codes§	Frequency (%*)		
	Overall (N = 72)	CNA (N = 35)	EVS (N = 37)
**Fear of Experiencing Another COVID-19 Outbreak**			
Fear of COVID-19 Coming Back into Facility	33 (46%)	17 (49%)	16 (43%)
*Fear of COVID-19 Coming Back into the Facility (No Cause Specified)*	*16 (22%)*	*6 (17%)*	*10 (27%)*
*Fear of Visitors/Family Bringing COVID-19 into the Facility*	*15 (21%)*	*10 (29%)*	*5 (14%)*
*Fear of Staff Members Bringing COVID-19 into the Facility*	*2 (3%)*	*1 (3%)*	*1 (3%)*
Hopefully, We Have a System in Place to Prevent COVID-19 Again	14 (19%)	6 (17%)	8 (22%)
Complacency in COVID-19 Prevention Practices	12 (17%)	6 (17%)	6 (16%)
*The General Public/Everyone*	*4 (6%)*	*2 (6%)*	*2 (5%)*
*Nursing Home Visitors and Family*	*3 (4%)*	*0 (0%)*	*3 (8%)*
*State*, *Local*, *Facility Restrictions Lifting*	*3 (4%)*	*3 (9%)*	*0 (0%)*
*Nursing Home Staff*	*2 (3%)*	*1 (3%)*	*1 (3%)*
COVID-19 Variants	7 (10%)	4 (11%)	3 (8%)
COVID-19 Differential (Is it COVID or Seasonal Allergies?)	1 (1%)	1 (3%)	0 (0%)
**Concerns About Mental Wellbeing of Staff**			
**Staff Emotional Strain and Anxieties**	10 (14%)	4 (11%)	6 (16%)
**Staff Feelings of Hopelessness–“This is never going to end”**	9 (13%)	3 (9%)	6 (16%)
**Staff Morale and Burnout**	5 (7%)	1 (3%)	4 (11%)
**Concerns About Mental Wellbeing of Residents**			
**Effects of Social Isolation (Increased Depression and Physical Deterioration)**	9 (13%)	7 (20%)	2 (5%)
**Lack of Care Due to Reduced Staffing**	1 (1%)	1 (3%)	0 (0%)
**Difficulty Managing Residents with Dementia**	1 (1%)	0 (0%)	1 (3%)
**Resident Feelings of Hopelessness–“Are we ever going to be able to see our families?”**	1 (1%)	0 (0%)	1 (3%)
**Concerns About Staffing Capacity and Future Workforce Development**			
**Staff Shortages**	5 (7%)	2 (6%)	3 (8%)
**Future Workforce Development and Capacity**	2 (3%)	1 (3%)	1 (3%)
**No Concerns Moving Forward**			
**I’m Not Concerned/Worried**	4 (6%)	2 (6%)	2 (5%)

* All responses were provided voluntarily and, due to the open discussion format, not every participant provided a response to every question. All summaries of qualitatively coded data are based upon only those responses received throughout the discussion. Therefore, percentages presented are calculated based on the total number of participants that provided a response to the individual question, not the total number of focus group participants. Participants’ responses were categorized to each individual code only once, however, responses may be categorized to more than one code. Therefore, percentages within a question may sum to over 100% and may not sum to the percentages for broader convergent themes presented in text. No statistical tests to compare responses by CNA and EVS participants were performed.

**§** Select quotes which operationalize each code may be found in the (S4 Table).

### Methods of communication for information about COVID-19

When responding to a Zoom poll question regarding where they go to find information about preventing COVID-19 in nursing homes, the majority of participants selected through their nursing home facility (63%), followed by the CDC website (19%), their state or local public health department (9%), the news (6%), their contracting agency (2%), and their coworkers (2%). Additionally, when asked how CDC can best reach staff with new guidance or information about COVID-19, most selected through their nursing home facility (64%), followed by a direct email from CDC (25%), a CDC webinar (4%), state or local public health department (3%), direct mail from CDC (2%), social media (1%), and through their professional organization or society (1%).

## Discussion

The objective of this qualitative assessment was to understand the impact of COVID-19 among nursing home staff and how both individual and facility level factors may have played a role in the pandemic experience. To explore such factors, we examined CNA and EVS staff perceptions of COVID-19 prevention efforts and self-reported behaviors and beliefs. Convergent themes and perceptions reported across the focus groups included the problem of staffing shortages, the toll of the pandemic on staff emotional and psychological well-being, concern for the physical and emotional well-being of nursing home residents, and the lack of consistent and systematic guidance resulting in rapidly changing infection prevention protocols. Additionally, the need for directly engaging CNAs and EVS staff members became evident, as many participants were grateful for the opportunity to participate in the discussion and shared invaluable insight through the lens of frontline staff members.

Participants across focus groups consistently reported the need to mitigate staffing shortages. Concerns included, but were not limited to, low wages and inconsistent employment benefits and incentives, such as supplemental hazard pay for essential workers. Participants shared the demands of taking on entirely new responsibilities and an increasing resident-to-staff ratio as nursing home staff quit or were placed under quarantine, and hiring new staff proved difficult. Staffing concerns have been similarly reported by frontline staff in previous studies [10, 11] and align with an analysis of NHSN data finding that nursing homes across the US have experienced significant staffing shortages during the COVID-19 pandemic [2]. Improving the employment outlook of CNA and EVS nursing home staff members may help ensure nursing home capacity and employee retention, as participants reported quickly burning out under the added pressure of an ongoing pandemic. Empowering CNAs has previously been associated with increased staff retention in US nursing homes [12] and a qualitative study with CNAs found the availability of resources, such as equipment and staffing, may help to enable coping with the increased emotional burden of the COVID-19 pandemic [13]. One observation of note from our findings was that when a participant stated they had received hazard pay, or other financial incentive for working during the pandemic, they tended to make more positive statements about their facility and its overall handling of the pandemic. Additionally, improving pay and benefits such as paid sick leave may be especially important, as even prior to the COVID-19 pandemic, a survey of staff working in long-term care facilities found that 70% of respondents reported feeling obligated to work while sick and almost 20% of CNAs held a second job [14].

Another resounding theme that emerged amongst the participants was the toll the pandemic took on the emotional and psychological wellbeing of CNA and EVS staff members, as well as concern for the physical and emotional well-being of the nursing home residents in their care. Due to prolonged isolation and restricted visitation in the nursing home, CNA and EVS staff described filling a familial role for residents, increasing both responsibility and emotional burden on an already taxing workload. CNA and EVS staff members emphasized their dedication to their residents, describing them as family, feeling heartbroken and helpless to improve their often-perceived hopeless situation. Both CNA and EVS staff reported feeling unprepared to handle the stress of their position in a pandemic that often felt never-ending. As this theme of stress and burnout has also been reported in other qualitative studies of nursing home staff [10, 11], mental health services may be important for nursing home staff affected by the close and compassionate role they serve for residents in nursing home care.

Participants also repeatedly described a lack of consistent and systematic guidance resulting in rapidly changing facility infection prevention protocols. Despite this, many felt their facilities did the best they could with what they had to support their staff, and that teamwork was an integral part to their collective survival through the COVID-19 pandemic. In the end, the focus groups became a space where participants could process the trauma of the pandemic amongst their peers and voice their invaluable perspectives on what went right and wrong through the lens of frontline nursing home staff. Participants expressed their gratitude for being included in this discussion, and for the space they were given to safely discuss their experiences.

This qualitative assessment was subject to several limitations. Participating nursing home facilities and staff represented a voluntary convenience sample. All data collected were self-reported and subject to recall bias, as well as social desirability bias. Generalizability of participant perceptions may be limited, as participants may not be representative of the overall nursing home staff population in the United States, with more participating facilities located in counties in the low/moderate range of social vulnerability and more facilities having non-profit ownership than the general population of non-participating US nursing homes. A higher percentage of focus group participants also identified as White compared to an analysis of long-term care staff nationally (68% versus 52%) [15] and a higher percentage of participating CNAs reported vaccination compared to aides in an analysis of nursing homes reporting vaccination coverage to NHSN as of March 1-April 4, 2021 (62% versus 46%) [16]. Additionally, generalizability may also be limited due to the small number of responses for some discussion questions and because outreach was conducted though nursing home administrators who facilitated staff member participation. All focus groups were conducted using the Zoom platform which posed unique challenges with varying Internet bandwidth and a learning curve with less technologically adept participants. In addition, due to the voluntary and anonymous nature of select data sources, the ability to measure associations between individual demographics (e.g., race, age, gender, COVID-19 infection status) and responses was limited; it may be of future interest to further explore potential relationships between individual and facility factors and participant responses in larger sample sizes. Future assessments should also examine staffing shortages and the impact of geographical location, SVI, facility ownership, and facility bed sizes on staffing ratios in a non-pandemic setting.

Despite these limitations, the focus group discussions illustrated that the overall impact of the pandemic was not simply whether a nursing home staff member tested positive for COVID-19, but rather the effect the pandemic had on the entire lived experience of these participants in both a professional and personal capacity. Addressing concerns of low wages and lack of financial incentives may have the potential to attract and retain employees to help alleviate nursing home staff shortages. Furthermore, access to mental health resources could help CNA and EVS staff cope with the emotional burden of the COVID-19 pandemic and increase resiliency. Additionally, CNA and EVS staff may benefit from training to improve their ability to care for residents’ emotional and psychological well-being. Speaking to these frontline staff members provided invaluable insight. Moving forward, CNAs and EVS staff should be a direct target audience for messaging of guidance changes. These frontline staff members should be included in improvement efforts to support nursing homes recovering from the impact of COVID-19 as well as future pandemic planning at the facility, state, and national levels.

## Supporting information

S1 Fig Zoom poll responses: How at risk were you of getting COVID-19 in your facility?. (PDF)

S1 Table Qualitative codes operationalized by select quotes from discussion: How have your job responsibilities or duties changed because of COVID-19?. (PDF)

S2 Table Qualitative codes operationalized by select quotes from discussion: What can your nursing home improve on? what is one thing you wish your nursing home could have done for CNAs/EVS staff during the pandemic to make things better?. (PDF)

S3 Table Qualitative codes operationalized by select quotes from discussion: What do you wish you would have known? what one piece of advice would you share with another CNA/EVS staff member about COVID-19 in nursing homes?. (PDF)

S4 Table Qualitative codes operationalized by select quotes from discussion: Moving forward, what are you most worried/concerned about related to COVID-19 in the nursing home? what do CNA/EVS staff need most moving forward?. (PDF)

S1 File Script of qualitative discussion questions and poll questions. (PDF)
