# Supplementary figures and images for: A qualitative assessment of factors affecting nursing home caregiving staff experiences during the COVID-19 pandemic
Source: PLoS One. 2021 Nov 15;16(11):e0260055. doi: 10.1371/journal.pone.0260055 (PMC8592470; doi:10.1371/journal.pone.0260055)

S1\_Figure.

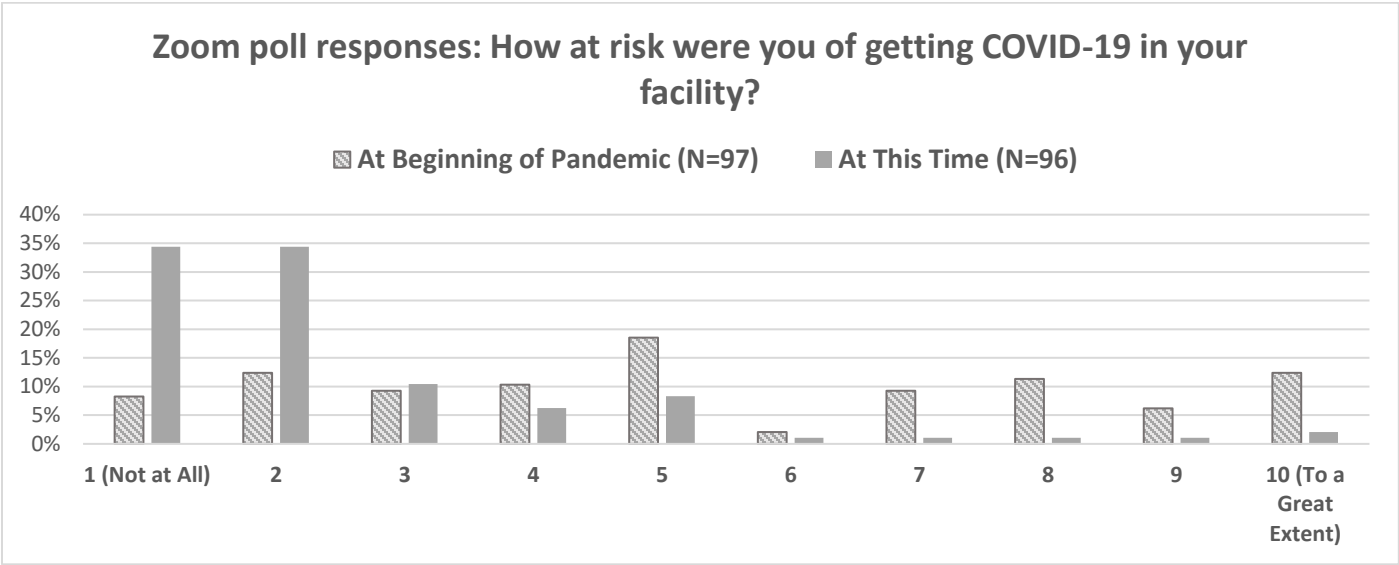

Supplement: S1 Fig — (PDF) [file pone.0260055.s001.pdf]
