# Supplementary material for: A qualitative assessment of factors affecting nursing home caregiving staff experiences during the COVID-19 pandemic
Source: PLoS One. 2021 Nov 15;16(11):e0260055. doi: 10.1371/journal.pone.0260055 (PMC8592470; doi:10.1371/journal.pone.0260055)
Supplement: S1 Table — (PDF) [file pone.0260055.s002.pdf]

S1 Table. Qualitative codes operationalized by select quotes from discussion: How have your job responsibilities or duties changed because of COVID-19?

Code	Operationalization
**Tasks Beyond Scope of Work and Added Responsibilities**	
Tasks Beyond Scope of Work	
**Rule and Protocol Enforcement (Screening, Distancing, etc.)**	• "It was really tough to keep everybody distanced from each other. It was a real tough skill. It was hard to keep everybody 6 ft apart." – CNA<br>• "Keeping them [residents] isolated from each other, it was really difficult especially in the beginning because we are all already in the facility, especially if their family members are out of town or out of state. And then telling them to separate when they've already built relationships with each other, it's really hard." – CNA
**Moving Residents Within Facility**	• "When someone would test positive, there was a crew of us, probably four or five of us that knew what to do, that gowned up, that would go in and actually move the resident. Take care of their laundry so it wasn't near anybody else. We would go in and clean the rooms, disinfect from top to bottom." – EVS
**Cleaning and Disinfection**	• "We had a lot of stuff added to us, housekeeping can't come to the hall and we had to help out there. We had extra work that we usually have people to do it. They couldn't come on, it was stressful." – CNA<br>• "We've actually increased [our work] because our dietary staff was no longer going on the unit, so housekeeping had to do cleanup in the dining areas for the residents' floor that were allowed out of the rooms and into the facility. So, we did have some increases [in tasks]. Just because of the reduction of staff." – EVS
**Non-Clinical Care of Residents Like Styling Hair or Bringing Food**	• "I was given a lot more to do. Like the hair shops were closed, I had to do their hair. I had to curl their hair. I actually worked in the COVID unit, so the kitchen wouldn't bring the food back to us, so we had to prepare [and] bring the food all the way back. So, a lot more was added on top to my plate, as well as taking care of the residents." – CNA
**Laundry**	• "In my facility we have in house laundry services and the laundry department wouldn't come to the floor to either collect laundry or to distribute the clean linens. As a CNA, we had to get all the clothes together and send them down. And when they would come back to the unit, they would just kind of put them on the elevator to our unit. And we would have to collect them and take them to the patients' rooms." – CNA
**Responsible for Patients Transferred from Overflowing Hospitals**	• "Unfortunately, when the hospital overflows, they send it to us. We actually had a point where we had one hallway shut down for people coming from the hospital." – CNA
**Administering Medications and Providing Treatments to Residents**	• "I was the with the first COVID patient here at our facility, the first positive result. At the time, LPNs were not comfortable coming into the room, and because they had trusted me, they had me giving medication and me doing treatments as well. So, a lot of things I wasn't responsible for before, they had trusted me to do with no issues. Done things I had never done as a CNA." – CNA
**Transporting Residents Outside of Facility (Medical Appointments)**	• "I actually at my job do several different things, so my job has changed quite a bit. Because I was also doing transport with residents for doctors' appointments, and I do medteching and CNAing. So quite a bit changed." – CNA

Code	Operationalization
**Transporting Deceased Residents to Morgue**	• "We actually had a transport team to where we didn't even allow the funeral homes to come in. We had two teams, and you were on call for a week and any time you had a death, we transported the bodies out to our morgue and the funeral homes picked them up there. We didn't want the funeral homes in, because we didn't want their dirty wheels, we didn't want their equipment in here that might have COVID and we didn't even allow them to come in, so we had to do that." – EVS
**Maintenance**	• "When we was having it [COVID], it did [change] because our maintenance man got sick so I was cleaning rooms, putting up the walls." – EVS
**Temporary Course to Assist Aides with Residents**	• "Oh yes, it increased a lot. I also did a temporary CNA course, so I was able to jump in and help the aides with the residents. We all worked together." – EVS
Added (Increased) Responsibilities	
**Additional Cleaning and Disinfection of High-touch Surfaces**	• "Yes, a lot, a lot more cleaning." – CNA<br>• "Absolutely. Just a lot of extra decontamination, using different cleaners and a lot of extra supplies, and a lot of wiping down. Just extra cleaning." – EVS
**Additional Laundry**	• "Oh yeah, that's definitely more time because you have to gown up and the laundry is just insane. Just trying to keep everybody in laundry is not easy." – EVS
**Increase in Time Required to Complete Tasks**	
**Additional PPE or Frequent Donning and Doffing Required**	• "I had to wear the N95 plus surgical masks, geared it up all the way. The whole 9 yards. When I wasn't one of the aides for the virus, we still had to wear the masks." – CNA
**Staff Shortages**	• "Well in the beginning it was a lot tougher, because a lot of people who was afraid, you know afraid for themselves and their families, so some people quit. Some people said it [COVID] was at the facility and they walked out. So, we had to all work together to make sure all the residents was taken care of." – CNA
**Taking More Precautions**	• "We do the precautions more, we follow the precautions very carefully." – CNA
**Frequent Changes in Protocols/Infection Control Procedures**	• "Some days, depending on our case situation, the policies would change. You just had to make sure you were in that huddle in the morning to get the information that you needed (to know what you would have to wear)." – CNA<br>• "The guidelines and rules changed daily. It was different every single day you came in. It was really hard at first because you could be wearing the right mask and all of a sudden, you're not wearing the right mask or you're doing the right procedure and that's wrong. It just really changed daily." – EVS
**Increased Workload Overall**	• "More work. More responsibilities." – CNA
**Longer Hours or Shifts**	• "I was working almost 60, 70 hours a week, I feel like I lived here most of the time." – EVS<br>• "It was very difficult, working 12, 16-hour days, very long. It was just chaotic; it was very stressful." – CNA
**Everything in General Takes Longer**	• "Job responsibilities itself haven't changed but the protocols or measures take longer, and it's being more careful in everything we do [that] has changed." – EVS
**More Handwashing**	• "We do have to be more careful with the handwashing, so it's just really more time consuming I would say." – CNA

Code	Operationalization
**Responsible for More Residents**	• "I was down on the COVID unit as well and we didn't have housekeeping down their either. Everything was kind of put on the CNAs during COVID. There was no housekeeping, kitchen, [CNAs] brought it down. There was a time when everybody was dropping like flies, so I was the only one down on the COVID unit to get everybody up. To give them their meds because I'm a MedTech. To feed them, to change them. It was just very hard." – CNA
**Added Pressures**	
**Increased Stress and Anxiety to Job**	• "I'm just going to put it right out there. It's been awful. It was an awful year; it was very scary. We didn't have everything that we needed in the beginning. The news of course you know in nursing homes, every single room has a TV and nonstop news all the time of COVID being fed to us while at work, to the residents." – CNA<br>• "It was a very stressful time in my experience. Asking the staff to do things that everybody is scared of doing, that was just stressful itself."– EVS
**Becoming Like Family to Residents**	• "We have been like their family, we've had more responsibilities of making the residents feel more comfortable without their families. And we've tried to do things to help the residents feel less neglected by their families. We have windows, we have phones, the families can come to the windows, they can talk to them on the phone, but most residents have voiced that they can't touch their families. That's sad." – CNA
**Consideration of Residents' Mental Health**	• "More thoughtfulness on residents' mental health too because that's a big issue. They are quarantined all day in their rooms without visitation for family members." – CNA
