# Supplementary material for: A qualitative assessment of factors affecting nursing home caregiving staff experiences during the COVID-19 pandemic
Source: PLoS One. 2021 Nov 15;16(11):e0260055. doi: 10.1371/journal.pone.0260055 (PMC8592470; doi:10.1371/journal.pone.0260055)
Supplement: S2 Table — (PDF) [file pone.0260055.s003.pdf]

S2 Table. Qualitative codes operationalized by select quotes from discussion: What can your nursing home improve on? What is one thing you wish your nursing home could have done for CNAs/EVS Staff during the pandemic to make things better?

Code	Operationalization
**Improve Staffing**	
**Mitigate Staffing Shortages**	• "I would like to say that everybody has agreed that we have shorter staff, there was nobody applying, nobody wanted to work and come in and get sick. We were trying to find help, but there was a lot of people afraid to come in and help, or work in healthcare right now. It's a problem in my facility." – EVS
**Provide Incentives or Hazard Pay**	• "Everyone, all my fellow CNAs have discussed about it. We feel that we got neglected with the hazard pay and no one wanted to come to work, because the pay. We felt like we had to do 10 times more work and we weren't being appreciated." – CNA<br>• "Well I don't think that the housekeeping staff been getting bonuses, so that could have helped." – EVS
**Hire In-House Staff (Instead of Agency Staff)**	• "Using agency staff throughout the home had really hurt because these people [were] from a bunch of different [nursing] homes, going to and from multiple [nursing] homes. I think that we had an outbreak, the first time we had one was in November. And, we think, it started from an agency staff who was in a different [nursing] home which had an outbreak." - EVS
**Improve Infection Prevention Practices**	
**Limit Constant Changes in Protocols and Guidelines**	• "Just the organization of it all was a big cluster. From day to day, we were told CDC wants us to do it this way, then they want us to do it that way. It was just a big confusion." – CNA
**Enhance Visitor Screening**	• "I just feel that we opened our facility too quick for family members to come in right now. Because of the COVID still going around, because you really don't know who got the vaccine, I mean everybody has different side effects. Not always when you check their fever, if they don't have a fever it doesn't mean they probably don't have COVID. If family members are coming in, they [should] need to have a negative statement to get to the facility, at least weekly." – CNA
**Ensure Consistent Enforcement of Protocols Across Staff (e.g., Wearing PPE Correctly)**	• "I would say I just wish they would still take it seriously. I'm not going to say none of them but 75% of the staff, they didn't take it seriously. They would laugh at our infection control. She would try really hard to make sure your goggles were on, this is what you need to do. And people would just, put their goggles up at the top of their head or not wear a gown. I just feel like a lot of the staff was…they didn't believe it. Some of them still don't believe it and that right there was heartbreaking, because we literally had a really bad wave of it, and had residents passing from it, and they still would be like, "Oh, I still don't believe it." So that really just took an emotional toll on a lot of people as far as you know, what are we doing this for? Our infection control lady would be like, "What am I enforcing all these rules for?" when nobody's following it, and she would be in tears. So, I would say for everybody to just work as a team. The whole facility, not just her and administration. Just the whole facility work as a team to come together to be like, listen, you don't believe it's real, you still have to follow the rules." – CNA
**Ensure Regular, Adequate Cleaning and Access to Cleaning Products**	• "Just make sure they actually washing their hands and keeping the building clean and sanitized." – EVS<br>• "And not just doing itself but also logging what we have done, like the high frequency touch areas so we can provide that proof if state would come in that we do indeed, every couple of hours go through and do all high touch areas. Disinfect and then log off on it." – EVS
**Ensure Sufficient PPE**	• "One thing they could have done better is that they could have had more PPE, they had to reuse a lot of the PPE, washed it and reused it. And at times they didn't have enough masks to provide, so it was a lot of washing masks, sterilizing them, having to reuse them." – EVS

Code	Operationalization
**Implement Mask Wearing Sooner**	• "Maybe start [to] use the masks or the face shields earlier, to protect the workers." – EVS
**Increase Testing of Staff and Residents**	• "As soon as COVID started, they should have started testing everyone in the facilities." – EVS
**Provide Education/Training to Staff**	• "One thing they could have done better is that they could have had more training." – EVS
**Dedicate Staff To COVID-19 Units Only**	• "When it first started, if you're a CNA and you're working with COVID, you would only be with COVID patients all day. Regardless, even if it was only two patients. Now, we still have other residents too and the COVID patient, even though we do know to do them last and less frequently. Only go in there if you need to… only stick with COVID patients when we have them." – CNA
**Do Not Open for Visitation Too Soon**	• "I just feel that we opened our facility too quick for family members to come in right now. Because of the COVID still going around, because you really don't know who got the vaccine." – CNA
**Restrict Visitors Earlier in Pandemic**	• "When we first heard about it, we didn't shut down soon. We still had guests coming in. So that could have prevented some." – EVS
**Improve Organizational Culture/Morale**	
**Improve Communication Within Facility**	• "Communicating things with us. You know, like say they would get the emails or whatever, just more communication on the whole COVID-19 virus." – EVS
**Don't Expect More Work Done in Same Amount of Time**	• "Between us and nursing, everybody has their own [work to be done]… but [we need] just a little more compassion. Saying this needs to be done now, we need to do this now. But when there's three other rooms that need to be fixed or changed or whatever, but it needs to be done now. And it's like, 'well let's just chip in and help a little bit'." – EVS<br>• "Expectations, that's it. Bigger, better, faster." – EVS
**Improve Organization and Preparation**	• "Well, our [physical] barriers [and preparation] weren't the greatest whenever we first got hit, but then our whole center ended up getting it, all the residents. We just needed to be better prepared on making our wall barriers that we had in place, because they didn't work out very well." – EVS
**Promote Teamwork and Accountability Among Staff**	• "The only real problem that I've had in the facility is just regarding masks [and accountability among all staff], like you'll see upper management not properly wearing their masks or shields walking through the building. And that's not really setting a good example for the rest of the employees and family members that come through." – CNA
**Show Appreciation and Compassion to Staff**	• "More communication and more appreciation for the ones who were willing to stay. And the ones that are still here with us. Just appreciation. Even just staff members telling me, 'I won't go to the COVID unit, they're not going to appreciate the work I do, so why am I going to go?'" – CNA
**Support Staff Despite Outside Pressures and Politics**	• "We kinda felt left out. We felt blame for the deaths. I know Governor [X] in [my state], put all the blame on us. And then at the end of the day, we never get compensated." – CNA
