# Supplementary material for: A qualitative assessment of factors affecting nursing home caregiving staff experiences during the COVID-19 pandemic
Source: PLoS One. 2021 Nov 15;16(11):e0260055. doi: 10.1371/journal.pone.0260055 (PMC8592470; doi:10.1371/journal.pone.0260055)
Supplement: S3 Table — (PDF) [file pone.0260055.s004.pdf]

S3_Table. Qualitative codes operationalized by select quotes from discussion: What do you wish you would have known? What one piece of advice would you share with another CNA/EVS staff member about COVID-19 in nursing homes?

Code	Operationalization
**Magnitude of Pandemic (Length, Seriousness, Transmissibility)**	• "That really, we're able to get it [COVID] anywhere. At first people were just only scared of going in the COVID-positive rooms, because they were positive there. But really looking back at it now, any one of us could have had COVID and not even known it." – CNA<br>• "I wish I would have known it was going to last so long. Nobody really knew." – EVS
**Use PPE**	• "Make sure you've got your PPE on. Appropriately, the right way. Make sure your mask is fitting right, make sure your goggles are fitting right, and your face shield and all of that." – CNA
**Treat Residents Like Family**	• "Take the extra time to help the residents because they need it most. They have no family. The families can't come, they can't spend time with them they can't do anything. We're an extension of their family. Any little thing you can do to help them feel better and keep them safe. Then do it." – CNA
**Wash Your Hands**	• "I would say, wash your hands, wash your hands… Washing your hands after every patient makes a big difference for preventing infections" – CNA
**Stay Vigilant**	• "I would say you can't let your guard down. You have to think about every little thing. It's the little things, that's where it can creep in." – EVS
**Follow IP Guidelines/Precautions**	• "Take the protocols seriously. Because I know a lot of people don't take some of the protocols we have seriously. It's just practicing infection control and keeping each other accountable with their infection control."– CNA<br>• "I would just say that anyone coming into the workforce, it is scary, it is real. But just as everything else, as long as you're taking the measures and precautionary steps to protect yourself and others, it will be just fine. It's just like fighting anything else. Just as long as your safe and you're protecting yourself in and outside the job." – EVS
**Have Emotional Resilience**	• "We were really put to the test; the CNAs have really gotten stronger throughout it. Just keep doing what we've been doing. Don't doubt yourself. It does get better. Be brave and believe in the good Lord, one day at a time." – CNA<br>• "Just keep your spirits as high as possible. They [the residents] feed on that energy too. I know it was tough, but we did it." – EVS
**Clean/Disinfect**	• "Just to make sure everything is disinfected because COVID-19 is real. And in the beginning, I think a lot of people didn't think it really truly was." – CNA<br>• "Clean well. That's really, that's what we can do in the nursing home. That's I think our biggest contribution was just make sure everything is sanitized multiple times a day. Through our outbreaks, we had a two-hour protocol, we had music go off and that meant all housekeepers get out and disinfect the hallways." – EVS
**Protect Yourself Outside Facility**	• "Stay home when not working and follow proper PPE, that's why I didn't get it [COVID]." – CNA<br>• "Definitely be safe outside of work. That's where I saw the biggest problem." – EVS
**Just Show Up**	• "I think just continue to do your job. Still show up, you're not needed any less, you're still needed if not more now and just do the proper thing that you need to do." – CNA<br>• "I think the one biggest thing that I would probably go back and tell myself is, you know, It's going to be stressful, like everybody says, but the job still needs to get done. We need to get COVID out of the building." – EVS

Code	Operationalization
**Take Care of Yourself/Stay Healthy**	• "Take plenty of vitamins, get plenty of rest, and stay healthy." – EVS
**Work Together**	• "You are one human being and you're not going to stop a pandemic by yourself. Lean on your team, because without your team, you're not going to accomplish this." – EVS
**Stay Home if Sick**	• "If you are sick, stay home. If you don't feel good, stay home. Don't take it [COVID-19] to your nursing home." – EVS
**Train, Educate, Prepare**	• "Going over your protocols, with your infection control doctors and nurses. Getting the right protocol and knowing how to start and what to do. I would say to get the right training." – CNA<br>• "I think the biggest thing is having the correct training on disinfecting, keeping things clean, even washing our hands. If everybody did that, I feel like COVID-19 wouldn't be spread as easily." – EVS
**Don't Move Residents Around**	• "We had COVID in our facility and it pretty much went through everybody, but at that point in time we were required to move the resident to an isolation room. And every time we moved them to the isolation room, I think there was more of a risk, because even if they was in a double room with another person, that other person got it anyway, so we should have left them in place. To me that was the biggest thing that spread it completely through our facility, we should have isolated them right away where they were at, instead of moving them to different locations." – EVS
**Warn that Supplies Would Run Out**	• "With the supplies, I didn't know we were ever going to run out of supplies like the mask, and the PPE. We had it all, we had it on, and then, all of a sudden, we didn't have anymore. If we would have known ahead of time, we would have never gone through shortage of the gloves and the mask and all that." – EVS
**Buy Stock in Alcohol**	• "To buy stock in alcohol." – CNA
**Facilities Shouldn't Overreact, Overdo It**	• "In my opinion, I think they [facilities] overdid it. Which I'm going to sound bad. But, [for example,] at the beginning we were throwing out everything. Anything that was touched by a person with COVID, their stuff was thrown out the door." – EVS
**Communicate (Have Adequate Communication)**	• "I would say biggest is communication. Make sure that you have adequate communication amongst the EVS staff, the laundry staff, management, and other departments." – EVS
**Stay on Floor if Working COVID Unit**	• "That once you enter the hot unit, you do not go off of that floor and interact with any other individuals, or any masked public areas like the lobby, where a lot of people seem to collect. I think that was our biggest problem when we had our outbreak, they should have stayed on their floors." – EVS
