# Supplementary material for: A qualitative assessment of factors affecting nursing home caregiving staff experiences during the COVID-19 pandemic
Source: PLoS One. 2021 Nov 15;16(11):e0260055. doi: 10.1371/journal.pone.0260055 (PMC8592470; doi:10.1371/journal.pone.0260055)
Supplement: S4 Table — (PDF) [file pone.0260055.s005.pdf]

S4_Table. Qualitative codes operationalized by select quotes from discussion: Moving forward, what are you most worried/concerned about related to COVID-19 in the nursing home? What do CNA/EVS staff need most moving forward?

Code	Operationalization
	**Fear of Experiencing Another COVID-19 Outbreak**
Fear of COVID-19 Coming Back into Facility	
Fear Of COVID-19 Coming Back into the Facility (No Cause Specified)	• "I pray to God we don't get it [COVID-19] no more."-CNA<br>• "That when people go on vacation, they'll bring it in. I worry about that the most." – EVS
Fear of Visitors/Family Bringing COVID-19 into the Facility	• "We aren't getting any positive cases right now because the majority of our patients are vaccinated, the majority of the staff is vaccinated, but the family members are the ones that I feel are the most trouble because they think because they've been vaccinated, everything is over now they should be able to come in the facility, touch, take off their masks, reach for a kiss. And it's still not the case. We're still having to talk to them and say, 'We still have to be cautious; we still have to be safe.' A lot of us here are really concerned with visitors. We are all so happy that visitors can come back in and our residents actually get to go out as well, that's exciting, but it's also scary. Because opening back up just opens us up for another outbreak." – CNA<br>• "If they are ready to open and let the family members come in and all kinds of residents, if they´re still not vaccinated, might bring the germs, might bring the COVID back." – EVS
Fear of Staff Members Bringing COVID-19 into the Facility	• "It's been a long time, and it's like most of us say, "Why don't we just open everything up and just let it go?" Because our residents are all negative. But it's the employees, we keep getting one employee here, one employee there, then a false positive and I don't know how to stop this." – CNA
**Hopefully, We Have a System in Place to Prevent COVID-19 Again**	• "Hopefully moving forward, we've got this, we have a system now, everyone here is tested once a week. Or if we have a case, we're tested twice a week. And everybody's using the PPE. We have a system now and moving forward it's just treating it like any other infectious disease, we just treat it with infection control." – CNA
Complacency in COVID-19 Prevention Practices	
The General Public/Everyone	• "What worries [me] moving forward is that a lot of people are still not taking care of themselves and they believe that now that they have the vaccine, they don't have to anymore, but they're still a lot of people who are not vaccinated and putting others at risk. So, more cases would be on the rise again as people are getting too comfortable." – EVS
Nursing Home Visitors and Family	• "Just it coming back into the facility, people are, you know, thinking they're Superman now that they are vaccinated and they can go on all these trips and then come and visit their loved ones." – EVS
State, Local, Facility Restrictions Lifting	• "I just hope that we keep some protocols in place. I'm in [City, State] so, [they announced] just today you don't have to wear masks anymore outside and I guess you don't have to wear them inside, as long as you're distanced enough away from people. But it's kind of scary because we've all been wearing the masks for so long, so now I just hope everybody keeps common sense." – CNA<br>• "I'm kind of worried that all the restrictions are going to be lifted. They're not really screening the people right and it's going to get more relaxed about everything and then it's just going to hit us real hard. That's probably what I'm most worried about." – CNA

Code	Operationalization
Nursing Home Staff	• "We all just have to worry about what we're doing outside of work, travel and social gathering, what we're doing when we're not here. As the weather gets warmer, we want to go out and have fun. I feel like we've all been couped up for the last year. That complacency factor that it's over, we're okay." – EVS<br>• "I feel like the staff has gotten very lenient with having their masks below their nose or not wearing the proper PPE. Not wearing their goggles. So, if we were to get it again, I think it would sift through the whole entire facility." – CNA
**COVID-19 Variants**	• "If I did have a worry it's that this strain mutates and we are going to be dealing with something different, maybe something worse."– CNA
**COVID-19 Differential (Is it COVID or Seasonal Allergies?)**	• "I would say that now that it's springtime and allergy season is here, a lot of people will have just sniffles and stuff like that and they automatically think, 'Oh it's COVID' and then they get tested and it's not COVID, so I think just all of the things that are seasonal that we deal with just as regular human beings makes us more afraid about that, even though it's just natural and it happens every year anyway." – CNA
**Concerns about Mental Wellbeing of Staff**	
**Staff Emotional Strain and Anxieties**	• "To have that emotional strain on us to continue to give the care that we give…you have to be on your "A" game. You have to be encouraging because they [residents] aren't going to get it anywhere else." – EVS<br>• "I think that's been the hardest thing for me is to see [the residents] suffer. I would do it all over again, I would move, I would do all the extra cleaning. But seeing them was the hardest." – EVS
**Staff Feelings of Hopelessness – "This is never going to end"**	• "I'm afraid things aren't going to get back to normal, the way it was." – EVS<br>• "I'm hoping this is the hardest it's going to be but that's my worry that this isn't the end. It seems like there's no end in sight. It's hard to be positive anymore. But we do it, that's our job." – CNA
**Staff Morale and Burnout**	• "Morale. You get run down cause we're asking them [staff] to do extra things for the same amount of pay. It's extra stress with all the set ups and tear downs and terminal cleans and it really shows on the staff that they're burnt out. And then again if you do get turnover, no one really wants to come in for a low paying job in the red zone you know. It's hard to get staff and it's hard to keep them happy when they're here. So that's my concern going forward." – EVS<br>• "I feel like a lot of staff members need a break. Whether that be a vacation at home. For us to continue to give the care that we give, we just need to breathe. I think this [COVID-19] really just needs to end at this point." – EVS

Code	Operationalization
**Concerns about Mental Wellbeing of Residents**	
**Effects of Social Isolation on Residents (Increased Depression and Physical Deterioration)**	• "I would say mental health of the residents. Many of my facilities, they cancelled their activities, they're quarantining in their rooms all day, they're not allowed to see family. Of course, with their mental health, their physical health also goes down, which is also a really big problem." – CNA<br>• "I worry for the residents. Sometimes when we test the residents, they don't want to get tested because they don't want to have a positive and be stuck in that room again. It really took a toll on them. I think it would be having an outbreak again, but for the purpose of the residents being hurt or just giving up." – CNA
**Lack of Care due to Reduced Staffing**	• "I just want to say through all of this, I think that's the major thing is the lack of care that has been able to be given to our residents. They have never been taken care of the same as they used to be since this [COVID] started, just because of working short all the time and the people that we're working with. And it's very heartbreaking. We can only do so much." – CNA
**Difficulty Managing Residents with Dementia**	• "With the residents, the main floors in our building… we had the dementia floor, which we lost a lot in, and then we have another floor, which they're all people that are hard to keep in their rooms. Cause they're wanderers, or they're the ones that touch each other a lot. They were our worst floors for keeping that [COVID] under control." – EVS
**Resident Feelings of Hopelessness – "Are we ever going to be able to see our families?"**	• "Well I guess what I need is kind of impossible to ask at this point, but I need this to end. Not just for me, but for our residents. They need to see their families. They need to have…we need to start coming to a normalcy again. I know things are never, probably never ever going to go back to the way things used to be prior to the pandemic. But [the residents] need to see a light at the end of this tunnel. I've had a couple of the residents say to me, "Is this ever going to end. Are we ever going to be able to see our families? Are we ever going to be able to touch our families and give them hugs?" Some of them have had grandbabies, great grandbabies be born. A lot of them get down and depressed and I've seen so much of that. They need to see a light at the end of the tunnel. When that question comes up, is there ever going to be an end to this, we can't answer that. We don't know. We don't know when this is going to end. They need a break from this." – EVS
**Concerns about Staffing Capacity and Future Workforce Development**	
**Staff Shortages**	• "Staffing. I live in a small town, so there really isn't nobody new to come and work. So, we have a lot of agency and I don't see that changing. People just don't want to get into long term care with COVID." – CNA
**Future Workforce Development and Capacity**	• "[My concern is] the type of employees that we continue to get [in the nursing home]. We don't have enough staff anymore. We can only do so much. A lot of people they would rather do a different kind of job than deal with all of this that we have to go through as caregivers with COVID. But the people that are willing to come, a lot of them, they go through schooling and they come, and they say, "I'm not doing this." They consider this crap. I'm not dealing with this. It's just too much. It just takes special people, some of us that have been doing this for years…. We knew why we were there, you know you're there to give care, no matter what the circumstance. But I don't think a lot of people coming into this industry or into this field have any idea what that involves, and, even to an extent of some of the people that had been working on this, they left and they quit because it's too much.. So, I'm hoping that in time, and my fear at the same time, is that we have more caregiving employees [in the nursing home] and training for them to be able to deal with that." – CNA
