# Supplementary material for: A qualitative assessment of factors affecting nursing home caregiving staff experiences during the COVID-19 pandemic
Source: PLoS One. 2021 Nov 15;16(11):e0260055. doi: 10.1371/journal.pone.0260055 (PMC8592470; doi:10.1371/journal.pone.0260055)
Supplement: S1 File — (PDF) [file pone.0260055.s006.pdf]

S1 File. Script of qualitative discussion questions and Zoom poll questions

Poll 1) How many years of experience TOTAL do you have as a CNA/Environmental Services (EVS/Housekeeping) staff member in a Long-Term Care Setting?

- 0-1 years
- 2-5 years
- 6-10 years
- 10+ years

Poll 2) How many years have you been employed as a CNA/EVS staff member at your CURRENT facility?

- <1 year
- 1-2 years
- 3-4 years
- >5 years

Poll 3) When do you most frequently work?

- Day Shift (during the week)
- Evening Shift (during the week)
- Night Shift (during the week)
- Weekends only

Poll 4) On a scale of 1-10, at the BEGINNING OF THE PANDEMIC, how at risk were you of getting COVID-19 in your facility?

Poll 5) On a scale of 1-10, AT THIS TIME, how at risk are you of getting COVID-19 in your facility now?

- Are CNAs/EVS staff working in a nursing home at risk for getting COVID-19?
    - Why or why not? (Probe: Job responsibilities)

- Have your (How have your) job responsibilities or duties changed because of COVID-19?
    - Have you had additional training as a result of COVID-19 and/or changed work responsibilities?

- Where do you feel more at risk of getting COVID-19?
    - Inside the facility where you work
    - Outside of the facility (NOT at work, at home, community)
    - Why? (Probe: perception of less control, if the perception has changed from one versus the other, what in the community or facility makes them feel safer)

- Has/how has the availability of the COVID-19 vaccine impacted how at risk you are of getting COVID-19?

- Thinking back --- What barriers/challenges to preventing COVID-19 existed in your facility <u>at the start of the pandemic</u>?

Now I would like to ask you another polling question -

Poll 6) At the BEGINNING OF THE PANDEMIC, what was the one greatest barrier/challenge to preventing COVID-19 in your facility?

Lack of training or education
Lack of Personal Protective Equipment (PPE)
Lack of leadership support
Staff disbelief (Staff NOT believing COVID-19 is a problem)
Staffing shortages
Frequent staff turnover
Limited COVID-19 testing
Other (Greatest barrier not listed above)
No barriers to preventing COVID-19 in facility

We have been through a year now (a year has passed since the beginning of the pandemic), if you had to pick the one greatest barrier/challenge to preventing COVID-19 in your facility, but AT THIS TIME, what would that be now? Currently? Today?

Poll 7) AT THIS TIME, what is the one greatest barrier/challenge to preventing COVID-19 in your facility now?

Lack of training or education
Lack of Personal Protective Equipment (PPE)
Lack of leadership support
Staff disbelief (Staff NOT believing COVID-19 is a problem)
Staffing shortages
Frequent staff turnover
Limited COVID-19 testing
Other (Greatest barrier not listed above)
No barriers to preventing COVID-19 in facility

  - For those who selected "Other", what was your one greatest barrier/challenge to preventing COVID-19?

- What barriers/challenges to preventing COVID-19 exist in your facility <u>currently/today/at this time</u>?

  - How have things (barriers/challenges) changed since the beginning of the pandemic? What helped you overcome these barriers/challenges?

- What has your nursing home done well throughout this pandemic?
- What can your nursing home improve on?

  - What is one thing you wish your nursing home could have done for you/your fellow CNAs/EVS staff during the pandemic to make things better?

- Do you believe you have a role in preventing COVID-19 in your facility?
    - How does what you do impact COVID-19 in the nursing home? For example, how do your daily activities at work impact COVID-19 in your nursing home?
    - How does what other CNAs/EVS staff, your coworkers, do outside of work (e.g., at home) impact COVID-19 in the nursing home? Activities outside of work, other jobs etc.?
- Looking back, what do you wish you would have known? What one piece of advice would you share with another CNA/EVS staff member about COVID-19 in nursing homes?
- Moving forward, what are you most worried/concerned about related to COVID-19 in the nursing home? What do CNAs/EVS staff need most moving forward?

Poll 8) Where do you most often go to find information on preventing COVID-19 in the nursing home?

My Nursing Home Facility
My Contracting Agency
My Union
Coworkers
State or Local Public Health Department
CDC Website
News
Social Media (e.g., Twitter)
Friends/Family
Other

Poll 9) Moving forward, how best can CDC reach CNAs/EVS Staff with new guidance or information about COVID-19?

Through Your Nursing Home Facility
Through Your Contracting Agency
Through Your Union
Through Your Professional Organization or Society (e.g., Association for the Health Care Environment etc.)
State or Local Public Health Department
CDC Webinar
Direct Email from CDC (e.g., newsletter)
Direct Mail from CDC (e.g., postcard)
Social Media (e.g., Twitter)
Other

- In terms of communication of new information about COVID-19, are there any other ways CNAs/EVS staff should be reached? Topics of interest or concern? (Probe: Messages or messengers?)
